# The College of Physicians in a New Character

**Published:** 1891-03-21

**Authors:** 


The Hospital

A Weekly Journal of

Science, Medicine, Nursing, and Philanthropy.

*For Hospitals, Asylums, Infirmaries, Dispensaries, Medical Practitioners, Students, Nurses, and the Charitable Public.*

Vol. IX.—No. 234.] [March 21, 1891.

### The College of Physicians in a New Character.

The President of the Royal College of Physicians of London occupies a position of dignity and some public influence. The position would have been much more dignified, and the public influence much greater and worthier if the history of the College had displayed more liberality and breadth of culture and a larger measure of public spirit. It cannot be denied that medicine in itself and in the persons of its ordinary practitioners is the most generous and kindly of the arts. It cannot be affirmed, without raising a smile, that those who have had the fortunes of the London College of Physicians in their hands for the last century or two have been models of large-mindedness, and worthy leaders and representatives of the scientific art which exists for the sole purpose of promoting human happiness by the means of human health. But the times and the manners are improving at the College, as well as elsewhere. The present President is both a man of the world and a physician of liberal instincts. If he could have his way the College would be the leader, the defender, and the inspirer of all that is broadest, wisest, justest, and most public-spirited in English medicine. Unhappily tradition dies hard; and when the tradition has been more than usually narrow and musty and small, it is almost impossible to kill it. Still, it is better late than never, and we wish the fellows and members of the College all the success which their new and most honourable departure merits.

Sir Andrew Clark, no doubt with the full concurrence of the more discriminating and generous of his confrères, has taken up the cause of that large class of loyal medical men who serve their country in her Majesty's armies. It is time that the most potential of the English medical corporations should show that it can place both a brain and a backbone at the service of the whole body of practitioners. Neither the medical profession nor the public knows very much about the doctors of the army. But what is publicly known is entirely to the honour of those medical soldiers. If space permitted we could tell many stories of medical men on the field of battle doing their duty to their wounded comrades whilst the bullets flew thick and fast around them. Has it ever been known that a doctor in the field has deserted a wounded man because his own life was in danger? British officers, and all British soldiers, we know, are brave men; but there are no officers or men who can be more safely relied upon to stand firmly by their duty in times of danger than the medical officers of the army. We do not claim more for them than their deserts: but why should we claim less? In this country every man wishes to see justice done; justice to every public servant.

The medical officers of the army have serious grievances, and the medical corporations, with the London College of Physicians at their head, have at length taken the field on behalf of their members. The general public is interested in this question exactly as it is interested in the question of the content or discontent of any other class of public servants. The public servants with which we are all most immediately familiar are postmen and policemen. It is highly important for us that policemen and postmen be well and fairly treated by the heads of their departments. When such public servants are well and fairly treated, their positions are sought after by the choicest of the the working-class population. The public is then well protected by the police, and well served by the postmen. These cases are exactly parallel with that of the army medical service. We want for the army, medical men of the highest professional and general culture, of the greatest readiness of resource, and of the kindest and worthiest character. We have but to imagine ourselves on a modern battlefield with the wounded, the dying, and the dead lying thick around us; and to see the deadly injuries inflicted on every vital part of the body by shot and shell, and piercing steel, and then we shall at once understand why some people think that a medical man of doubtful character and deficient professional capacity ought rather to be shot by court-martial than to be permitted to deal with the wounded and the dying who have fought in their country's cause. The policy of the heads of department which drives away the best medical men from the army and appeals only to the lowest, the most incompetent, and the least worthy members of the profession, is a policy of danger and death to the fighting soldier, of loss and injury to the public; and, therefore, of madness which is little less than criminal.

What is it that the medical officers of the army complain of? They say that they are given titles without rank, and are exposed to dangers without honour. That, in short, they are treated as if they were in some way inferior to the combatant officers; and whilst they are always asked to share their dangers, they are only on very exceptional occasions invited to take part in their rewards. We happen to know that these charges are literally true, otherwise we should be inclined to disbelieve them. Moreover, it has been the writer's experience to associate intimately with a good many officers of the army, and with pupils of Sandhurst and Cooper's Hill, and of the different army tutors, as well as with medical practitioners and medical students. Speaking as an insider he is amazed at two things:

first, at the assurance of the combatant officers of the army which inspires their objection to the claims of the medical officers; and secondly, at the rank folly of any well-educated and well-qualified young medical man who thinks for a moment of seeking service in the British army under present conditions.

The combatant officers are not without an excuse which looks plausible when they object to the medical officers holding substantive rank and being treated in every other respect exactly as the combatants are treated. They say that "To incorporate the medical staff into the general army, and to give its officers substantive rank and military titles would have the effect of placing a medical officer in military command over troops in the field whenever one might happen to be the senior officer present." There is a show of reason here, and if the case were as stated, it would be a not irrational objection. But Sir Andrew Clark, in his letter to Mr. Stanhope, proves to demonstration from the various books of regulations that the plea is absolutely without foundation of any kind. As Sir Andrew says: "I have conclusively proved from the Royal Warrant of 1890 that this objection has no valid existence, and that definite provision has been made for the granting of rank and title in special circumstances without command; and I may be permitted further to remind you that at this time there are to be found in the army numerous officers of almost all ranks who have no combatant command." Well may Sir Andrew add these weighty words—"It seems to me that to deal with an official having responsibilities of such moment as if he were merely a doctor, as if he were not really an officer, as if he were not entitled to rank as combatant's rank, and as if he were not worthy of being incorporated into the general army, is at once a serious anomaly, a tactical mistake, and a grave injustice."

This is really a question for the public, even more than for the heads of departments. The medical officers of the army are public servants, and the heads of departments are public servants too. The other day a member of Parliament complained that the clerks of the House of Commons had got into the way of acting as if they were the masters of the members, and not their servants. So much the greater fools are the members to have allowed such a state of things to come to pass. But exactly the same thing seems to have taken place with those heads of departments who are responsible for the administration of her Majesty's armies. Sir Andrew Clark addresses Mr. Stanhope in language which is mild. But the College of Physicians is fairly roused, and it cannot have a better fighting head than its present President. Will it throw away the wrappages of a somewhat cumbrous and antiquated professional dignity, and fight like a veteran regiment at the head of the other medical corporations? If it will, they will follow; and not only they, but every medical practitioner in the kingdom who has measured brains and culture with a combatant military officer. As a matter of fact, the military officers of the present day have acquired their peculiar status by having the courage—or shall we say the assurance?—to ask for it, and to take it. If the medical officers of the army are to acquire that position which is theirs by right, they must not be slow to follow the example set them by their brothers in arms.

